# The test-retest reliability of the Opiate Treatment Index in nyaope users in Johannesburg

**DOI:** 10.4102/sajpsychiatry.v30i0.2087

**Published:** 2024-02-16

**Authors:** Kelebogile Pitsoane, Nirvana Morgan, Sumaya Mall

**Affiliations:** 1 Department of Psychiatry, Faculty of Health Sciences, University of the Witwatersrand, Johannesburg, South Africa; 2 School of Public Health, Faculty of Health Sciences, University of the Witwatersrand, Johannesburg, South Africa

**Keywords:** nyaope, opiates, test-retest reliability, opiate treatment index, substance users, opioids, heroin, rehabilitation, Johannesburg

## Abstract

**Background:**

Epidemiological studies suggest that nyaope, a heroin-based drug, is widely used in South Africa. Yet few reliable research tools are available to assess treatment outcomes of users. The Opiate Treatment Index (OTI), a tool developed in Australia, could potentially facilitate research on context-specific South African treatment outcomes. However, we know little of its test-retest reliability.

**Aim:**

This study aimed to assess the test-retest reliability of the OTI among a sample of nyaope users in Johannesburg.

**Setting:**

This study was conducted across three substance use treatment facilities in Johannesburg.

**Methods:**

The OTI was administered to 53 nyaope users at baseline and one week later. To determine the test-retest reliability of the OTI, the intra-class correlation coefficients (ICC) and the Brennan–Prediger coefficients of the two interviews were calculated.

**Results:**

The ICC of the Q-scores from the data sets along with the Brennan–Prediger coefficient for the substance use domain were calculated. The ICC for nyaope was 0.38. Brennan–Prediger coefficients were as follows: alcohol – 0.96, crack-cocaine – 0.89, cannabis – 0.92, methaqualone – 0.85 and crystal methamphetamine – 0.89.

**Conclusion:**

A significant positive finding was the excellent test-retest reliability of the injecting and sexual behaviour domains and moderate reliability of the criminality, general health and social functioning domains.

**Contribution:**

The results of this study provide insight into the reliability of this tool and for its use in future studies in the South African context.

## Introduction

The South African Stress and Health Study (SASH) suggests that the lifetime prevalence of substance use disorders in South Africa is as high as 13%.^1^ With regard to the use of more specific substances, the South African Community Epidemiology Network on Drug Use (SACENDU) reported that heroin is the third most commonly used substance in South Africa and the most commonly used substance in patients presenting for substance use treatment.^2,3^ Furthermore, research suggests that heroin use can be associated with both psychopathology and reduced quality of life.^4,5,6^ According to the report by SACENDU,^2,3^ about 4% of Gauteng substance users report using nyaope (a street drug that consists of heroin as a principal ingredient) as their primary substance.^2^ Data from adolescent and adult samples in South Africa suggest that the prevalence of nyaope use is 11.5% among adolescents and that the majority of nyaope users are males of African ethnicity.^2^

A World Health Organization (WHO) study of heroin users in six low- to middle-income countries (LMICs) utilised the Opiate Treatment Index (OTI), a comprehensive instrument, to examine the prevalence of substance use.^7^ The OTI is a clinician-administered structured interview that was developed in Australia. It measures the effectiveness of drug treatment through six treatment outcomes, namely human immunodeficiency virus (HIV) risk-taking behaviour, social functioning, criminality, health status and psychological functioning. Research from Australia, the United Kingdom (UK), Spain and China, a combination of high- and low- to middle-income countries have examined the OTI in relation to treatment outcomes for opiates, heroin and other substances.^8,9,10^ The OTI incorporates change in drug use, injecting and sexual practices, social adjustment, criminal history and psychological adjustment.^11,12^ These domains and the scope of the tool suggest potential for examining treatment outcomes both prospectively and comprehensively.^12,13,14^

In South Africa, a few studies have employed the OTI. Morgan and colleagues,^13,14^ who used the OTI on a sample of heroin users in Johannesburg to examine treatment outcomes, suggest that the OTI may be useful and potentially adaptable in the South African context. However several key questions about the utility of this instrument remain unanswered. These include questions about the reliability of the OTI, which has not been examined in the South African context. The reliability of a tool entails measuring the consistency of results when used in a similar context at different points in time and when used by different investigators. More specifically, the test-retest reliability refers to how well an instrument can accurately reproduce similar results when measuring the same variable(s) at different points in time by the same investigator. The more reliable an instrument, the more likely it is to produce consistent results and less likely to have the results affected by human error.^15^ Little is known about the test-retest reliability of the OTI in the South African setting.

## Objectives

The first objective was to measure the test-retest reliability of the drug use, criminality, general health status, injecting and sexual risk-taking behaviour and social functioning domains of the OTI in a South African sample of nyaope users in Johannesburg area.

The second objective was to describe the demographic and clinical characteristics of nyaope users admitted to the three designated rehabilitation facilities, namely iThemba Drug and Alcohol Rehabilitation Clinic, Life Rehabilitation Centre (LRC) and Empilweni Rehabilitation Centre. The characteristics that were described include the ethnicity, age, marital status, employment, level of education, living arrangements, HIV status and past month substance use.

## Research methods and design

### Study design

This was a quantitative, prospective study.

### Setting

The participants were recruited over a period of 5 months, from October 2019 to February 2020. The setting included three state-funded rehabilitation centres across the greater Johannesburg areas.

Participants were recruited from the iThemba Alcohol and Drug Rehabilitation Clinic in Krugersdorp, which has a capacity for up to 40 male users. Life Rehabilitation Centre is situated in Randfontein and has capacity for up to 200 male and female users. These rehabilitation centres offer voluntary inpatient treatment to substance users seeking inpatient detoxification and psychosocial rehabilitation. Only two participants were recruited from Empilweni, which is a rehabilitation centre in Soweto.

### Participant recruitment

Convenience sampling was employed to recruit the sample. Staff members at the respective facilities informed the principal investigator (PI) when nyaope users were admitted to the facilities. Nyaope users were invited to participate in the study. Screening and recruitment of participants was conducted during the users’ first week of admission.

### Inclusion criteria

Potential participants were screened to ascertain that nyaope was their primary drug of use and that they were not experiencing any signs of withdrawal from nyaope. Nyaope users who were above the age of 18 and those who gave informed consent were included in the study.

### Exclusion criteria

Users who were medically unstable, that is, still going through detoxification and showing signs of withdrawal from substance use, and those who declined to participate in the research project were not included in the study. Recruited participants who left the rehabilitation facilities prior to completing the second interview were also excluded from the study.

Fifty-six individuals from all the rehabilitation facilities were screened. One of the screened individuals did not meet the inclusion criteria because he was medically unstable during his first week of admission and two users signed ‘refusal of hospital treatment’ (RHT) after the first interview, so they were excluded from the final sample. Thus, the final sample consisted of 53 participants, from three rehabilitation centres, who completed two interviews 7 days apart.

### Data collection

#### Instruments

Consent to use the OTI for research purposes was granted by the developer of the OTI, Professor Shane Darke.^11^ Permission was granted provided that the PI gave due acknowledgement to the developers.

Once participants gave consent to participate, demographic data were entered in a separate data collection sheet and the anonymity of the data was preserved. The demographic data collected included: (1) ethnicity, (2) age, (3) marital status, (4) employment, (5) level of education, (6) living arrangements and (7) HIV status. The OTI was also administered and data regarding the substance use in the past month preceding the interview, criminality, general health status, injecting and sexual risk-taking behaviour and social functioning domains was captured on a data sheet. The second interview was conducted 7 days after the first one for each participant. During the second interview, demographic data were not collected, only data from the domains of the OTI as mentioned here were collected.

At both interviews, the questionnaire posed questions about the same time frame, namely, the month prior to their admission at the rehabilitation facility. The interviews were approximately 20 min – 30 min long.

All the interviews were administered by the PI (K.P) a trainee psychiatrist. The participants did not complete any part of the questionnaire by themselves.

The OTI was tailored and amended to be more relevant to a South African setting, so as to minimise bias or error that may occur because of cultural differences between the population assessed by the originator of the OTI and the population in our sample.

The specific changes included the names of specific drugs and terms used for quantifying their use to reflect the common local street vernacular. For example, ‘number of hits of heroin’ on the original OTI was changed to ‘number of bags of nyaope’ in the amended version:

- South African colloquial term for beer, that is, ‘dumpie or quarts’ was included while describing units of alcohol consumed. This was also performed by investigators in the UK who had made minor changes to OTI to make it relevant to their own setting.^8^
- One other minor amendment was the change of the term ‘rarely’ to ‘hardly ever’.
- The psychological adjustment section was omitted from the amended OTI because the OTI developers used the General Health Questionnaire (GHQ) to assess psychological adjustment. The GHQ is an instrument that was developed in the 1970s. It is used to screen for minor psychological or psychiatric, but non-psychotic, disorders and its validity and reliability have already been established.^16,17,18^

### Sample size estimation

For the estimation of an intra-class correlation coefficient (ICC) of 0.80 for test-retest reliability, with a confidence interval width of 0.20, with 80% power, a sample size of 52 participants was required.^19^

### Calculating the Q-score of the opiate treatment index

The drug use section of the OTI asks the user to provide the number of drug use episodes on each of the past 3 days the drug was used. The number of days between substance use episodes was also captured. An equation was then used to calculate the frequency of drug use in the past month:

$$Q=\frac{q1+q2}{t1+t2}$$ Eqn 1

*Q* = average amount per day

*q*1 = amount consumed on the last use occasion

*q*2 = amount consumed on the second last use occasion

*t*1 = interval between the last day of drug use and the next to last use day

*t*2 = interval between the second and third last days of drug use.

The result of the calculation is represented as a Q-score, as shown in Table 1, which can be interpreted as follows: Q-score of 0.00 = past month abstinent, Q-score of 0.01–0.13 = uses once a week or less, Q-score of 0.14–0.99 = uses more than once a week, Q-score of 1.00–1.99 = daily use and a Q-score of 2 = uses more than once in a day. The drug use section of the OTI also allows for the analysis of the categorical variable of ‘any’ past month substance use versus abstinence. Thus, the Q-score represents a more detailed frequency of the drug used in the past month while ‘any use’ (Q-score > 0) versus abstinence (Q-score = 0) simply represents whether the participant reported any substance use in the past month.

**TABLE 1:** Interpretation of Q-scores.

Q-score	Frequency of use
0.00	Abstinent
0.01–0.13	Once a week or less
0.14–0.99	More than once a week
1.0–1.99	Daily
≥ 2	More than once a day

*Source:* Adapted from Darke S, Hall W, Wodak A, et al. The Opiate Treatment Index (OTI) manual [homepage on the Internet]. 1991 [cited 2021 Jul 02]. Available from: https://ndarc.med.unsw.edu.au/sites/default/files/ndarc/resources/TR.011.pdf

### Data analysis

Descriptive analysis of the study population was carried out as follows: Categorical variables were summarised by frequency and percentage tabulation. Continuous variables were summarised by the mean, standard deviation, median and interquartile range.

The ICC was used to assess the correlation and agreement of the data of the two interviews for the Q-score and all other domains of the OTI. The ICC is an index that measures the correlation and agreement between measured variables and is a suitable tool for test-retest reliability and therefore preferred to other correlation coefficients such as the Pearson correlation coefficient.^20,21^ The interpretation of the ICC and the Brennan–Prediger coefficient are presented in Table 2 and Table 3, respectively.

**TABLE 2:** Intraclass correlation coefficient interpretation.

ICC	Strength of agreement
< 0.5	Poor reliability
0.5–0.75	Moderate reliability
0.75–0.9	Good reliability
> 0.9	Excellent reliability

*Source:* Adapted from Koo TK, Li MY. A guideline of selecting and reporting intraclass correlation coefficients for reliability research. J Chiropr Med. 2016;15(2):155–163. https://doi.org/10.1016/j.jcm.2016.02.012

ICC, intra-class correlation coefficients.

**TABLE 3:** Brennan–Prediger coefficient interpretation.

Brennan–Prediger coefficient	Strength of agreement
< 0.00	Poor
0.00–0.20	Slight
0.21–0.40	Fair
0.41–0.60	Moderate
0.61–0.80	Substantial
0.81–1.00	Almost perfect

*Source:* Adapted from Landis JR, Koch GG. The measurement of observer agreement for categorical data. Biometrics. 1977;33(1):159–174. https://doi.org/10.2307/2529310

For a better understanding of the test-retest reliability of the drug use section of the OTI, we performed an additional analysis looking at substance use as a categorical variable of any past month use versus no past month use. In order to do this, we assessed whether a participant stated any use or non-use of a substance in the first interview and correlated it with their answer in the second interview. The test-retest reliability for the drug use section categorised as use versus no use was established by the Brennan–Prediger coefficient of agreement as depicted in Table 3. Data were managed using SAS version 9.4 for Windows,^22^ and a 5% significance level was used.

### Ethical considerations

The study was approved by the University of the Witwatersrand Human Research Ethic Committee. The ethics approval number is M190633. The centres granted the investigator permission to collect data at their facilities. The centres granted the investigator permission to collect data at their facilities. Participation in the study was voluntary. The participants were informed of their rights to refuse or to agree to participate and that their participation or a lack thereof would not in any way impact the treatment that they were receiving.^23^

## Results

### Sample demographics

The demographic details of the participants are presented in Table 4. The median age of the participants was 29 years (IQR 22–33 and range 20–40 years). Ninety-two per cent (*n* = 49) of the participants were male and 8% (*n* = 4) were female. Black African people made up 92% (*n* = 49) of the sample. Seventy-four per cent (*n* = 39) had a secondary school education although they had not completed high school. Thirteen per cent (*n* = 7) had completed high school only. Eight per cent (*n* = 4) had post-grade 12 qualifications. Fifty-five per cent (*n* = 29) were unemployed. Forty-three per cent (*n* = 23) were employed. All the participants were unmarried. Seventy per cent (*n* = 37) lived with their family. Thirteen per cent (*n* = 7) were living with friends. Nine per cent (*n* = 5) lived alone.

**TABLE 4:** Demographics of study participants.

Demographics	Total (*N* = 53)	Percentage
**Gender**		
Male	49	92
Female	4	8
**Ethnicity**		
Black African people	49	92
Mixed race people	1	2
Indian people	2	4
Caucasian people	1	2
**Level of education**		
Primary school	3	5
High school (Gr 8–11)	39	74
Matric (Gr 12)	7	13
Tertiary	2	4
Technical skill-based certificate	2	4
**Employment**		
Employed	23	43
Unemployed	29	55
Student/scholar	1	2
**Marital status**		
Single	53	100
Married	0	0
**Living arrangements**		
Living with family	37	70
Living with friends	7	13
Living alone	5	9
Homeless	4	8
**HIV status**		
Positive	9	17
Negative	39	74
Unknown	5	9

### Past substance use

The median age of onset of any substance use was 15 years old (IQR 13–17; range 9–20 years old). The median duration of substance use was 14 years (IQR 9–18; range 5–33 years). The median age of onset of nyaope use was 21 years (IQR 18–25 years; range 14–42 years), and the median duration of use of nyaope was 6 years (IQR 3–9 years; range 0–19 years).

Ninety-eight per cent (*n* = 52) of participants used cannabis, 96% (*n* = 51) used tobacco and 74% (*n* = 39) used alcohol prior to the onset of nyaope use. Fifty-seven per cent (*n* = 30) of participants had been admitted to a rehabilitation centre before their current admission. With regard to methods of nyaope use, 94% (*n* = 50) reported smoking nyaope with cannabis. Thirty-four per cent (*n* = 18) of the participants were injecting nyaope. Nineteen per cent (*n* = 10) were chasing (heating and inhaling the vapour) nyaope. Seventeen per cent (*n* = 9) stated they were HIV positive. Seventy-four per cent (*n* = 39) were HIV negative, and the remaining 9% (*n* = 5) did not know their HIV status as they had never had an HIV test.

#### Past month substance use (Q-scores)

The frequency of drug use in the past month (median Q scores) and the number of users reporting any substance use in the past month are depicted in Table 5 and Table 6, respectively. The median Q-scores for specific substances are: (1) tobacco was 9.4–10.2, (2) crystal methamphetamine 0.2–0.3, (3) crack cocaine 0.7–0.9, (4) nyaope 4.1–5, (5) cannabis 0.3–0.2, (6) inhalants – 0.1, (7) methaqualone – 0.1 and (8) tranquillisers 0.1–0.2. For inhalants, it was 0.1; for other opiates 0.8 and for alcohol 0.6–1.1.

**TABLE 5:** Frequency of substance use in the past month (median Q-scores).

Substance	Median Q-scores			
	First interview (*N* = 53)		Second interview (*N* = 53)	
	*n*	IQR	*n*	IQR
Nyaope	4.1	2.5–5	5	3–6.5
Cannabis	0.3	0	0.2	0
Tobacco	9.4	4–11	10.2	5–15
Crack cocaine	0.7	0–0.036	0.9	0–0.11
Crystal methamphetamine	0.2	0	0.3	0
Methaqualone	0.1	0	0.0	0
Inhalants	0.1	0	0.0	0
Tranquillisers	0.1	0	0.2	0
Opiates	0.8	0	0.8	0
Alcohol	0.6	0–0.036	1.1	0–0.036
Hallucinogens	0.0	0	0.0	0

IQR, interquartile range.

**TABLE 6:** Number of participants reporting any past month use (Q-score > 0).

Name of substance	First interview (*N* = 53)		Second interview (*N* = 53)	
	*n*	%	*n*	%
Nyaope	53	100	53	100
Tobacco	52	98	53	100
Cannabis	10	19	8	15
Crack cocaine	14	26	15	28
Crystal methamphetamine	8	15	9	17
Mandrax (Methaqualone)	10	19	8	15
Inhalants	1	2	0	0
Other opiates	2	4	2	4
Tranquillisers	2	4	2	2
Alcohol	15	28	14	26
Hallucinogens	0	0	0	0

### Test-retest reliability

#### Substance use section of the opiate treatment index

The ICCs as presented in Table 7 are as follows: the ICC of the Q-score for tobacco was 0.91, for crystal methamphetamine, 0.90, for tranquillisers, 0.92 and for other opiates, 1.00, which fell into the excellent category. The ICC for crack cocaine was 0.65, this was in the moderate range. The ICCs for nyaope (ICC = 0.38), alcohol (ICC = 0.28), cannabis (ICC = 0.42), methaqualone (ICC = 0.37) and inhalants ICC = 0) were in the poor range. Using the categorical variable of use versus no use in the past month, the Brennan–Prediger coefficients were as follows: crack cocaine – 0.89, for cannabis – 0.92, for methaqualone – 0.85, for crystal methamphetamine – 0.89, for other opiates – 1.00, for tranquillisers – 1.00, for inhalants – 0.96 and for alcohol – 0.66. For nyaope and for hallucinogens, the Brennan–Prediger coefficient could not be estimated as there were no non-users for nyaope and no users at all for hallucinogens. All substances showed a greater than 0.8 Brennan–Prediger coefficient, which is almost perfect agreement.

**TABLE 7:** Correlation coefficients of the opiate treatment index.

Substance used and domain of functioning	ICC†	Brennan-Prediger coefficient
Nyaope	0.38	not estimable – (no non-users)
Tobacco	0.91	0.96
Alcohol	0.28	0.66
Crack cocaine	0.65	0.89
Cannabis	0.42	0.92
Methaqualone	0.37	0.85
Crystal methamphetamine	0.90	0.89
Other opiates	1.00	1.00
Tranquillisers	0.92	1.00
Inhalants	0.00	0.96
Hallucinogen	Not estimable – no users	Not estimable – no users
OTI injecting subtotal	0.95	-
OTI sexual behaviour subtotal	0.81	-
OTI injecting and sexual behaviour total	0.94	-
Social functioning total	0.71	-
Crime total	0.61	-
Total general health score	0.62	-

† , lCC, intra-class correlation coefficient; OTI, opiate treatment index.

#### Injecting, sexual behaviour, criminality, social functioning and general health

The ICCs for the injecting subsection was 0.95 and sexual behaviour subsection was 0.81 (Table 7). The ICC for social functioning was 0.71, for crime was 0.61, for total health was 0.62, which is moderate.

## Discussion

This study aimed to assess the test-retest reliability of the OTI in nyaope users in Johannesburg, South Africa. The study used the ICC to assess the correlation of Q-scores of the domains under investigation and the Brennan–Prediger coefficient to assess the use versus (vs.) abstinence correlation of the substance use results. This study yielded several important findings. Using the ICC to assess the test-retest reliability of the following domains, that is, substance use, sexual and injecting behaviour, social functioning and crime and general health, variable test-retest reliability was found. While using the Brennan–Prediger correlation coefficient for the use versus abstinence for substance use, the test-retest reliability of the past month drug use section was found to be almost perfect for all substances excluding alcohol, which fell within the substantial category. The Brennan–Prediger correlation coefficient for nyaope could not be assessed, because all participants reported using nyaope at both interviews.

The injecting and sexual behaviour domain had an excellent reliability. In this sample, relatively fewer participants reported having sexual encounters in the last month. The majority of participants reported that these fewer encounters were largely because of low libido as a result of nyaope use. Also, because the majority of the study sample consisted of male users, there were fewer reports of dependency on an intimate partner for acquisition of substances and hardly any report of engaging in transactional sex or solicitation in order to obtain substances. Injecting behaviour also had an excellent test-retest reliability. These results may be attributable to the sensitive and discreet manner of the PI in ensuring that information of a sensitive nature was not shared with the treating facility and that the interviews were conducted in quiet and private rooms, thereby minimising any risk of breach of confidentiality. This discretion may have reduced the inclination to report inaccurately. The excellent reliability of the injecting and sexual behaviour was unexpected and yet encouraging, more especially considering the sensitive nature of this information and perceived stigma and shame that is associated with high-risk sexual behaviour. This demonstrates the potential of this particular section of the OTI for reliable data collection.

The general health, social functioning and crime sections showed moderate reliability. The questions regarding social functioning required recall of events 6 months prior and required recall of isolated incidents. With regard to self-reported past month criminal activities, there was an expected bias because of perceived stigma, social desirability and the sometimes highly sensitive nature of the crime reported. However, despite this, as with social functioning, the criminality section of the OTI demonstrated moderate reliability. The results of the sexual and injecting behaviour, general health, crime and general health domains produced results in keeping with previous similar studies wherein these domains also showed moderate to good correlation and reliability.

The ICC for use of other opiates, tranquillisers, tobacco and crystal methamphetamines showed excellent test-retest reliability. The Q-scores of the remaining substances used, namely cannabis, nyaope, methaqualone and inhalants had poor test-retest reliability. These results were in contrast to the results of other investigators, in the UK and China, who also assessed the test-retest reliability of the OTI using approximately 7–14 and 7–10 days between first and second interviews, respectively.^8,9^ The aforementioned international studies reported good test-retest reliability across all domains of the OTI when the interviews were conducted by both different and the same interviewers.^8,9,10,11^ To better understand the reasons for differences in results between previous international studies and our study, we considered variations in methodologies such as duration between interviews and statistical analysis as well as differences in baseline demographic factors between the samples.

The Australian and Spanish studies used 7-day intervals between interviews.^10,11^ The time interval between the data collection points is important. If this interval is too long, certain variables may have changed in that time. If too short, some results may be overestimated and inaccurate.^11,12^ During data collection for this study, the PI administered the tool to a sample of nyaope users at two time points, seven days apart, to replicate the conditions of the original Australian study as far as possible.^11^ It is also important to observe that the aforementioned studies are much older and utilised different statistical analysis compared to this study; therefore their results may not be fully comparable. Literature by Koo and Li^21^ and Landis and Koch^20^ recommended the ICC and Brennan–Prediger correlation coefficients as the most appropriate statistical test. Accordingly, we utilised these statistical tests in our study. Differences in the baseline demographics between the current study and previous ones were few. The median ages for the users in the British studies was 34.2 years while that of the Spanish study users was 30, which were fairly similar to the original Australian study.^8,10^ Additionally, in all studies, the majority of participants were male. The participants in the Chinese and Spanish studies had less than 8 years of schooling on average, compared with our sample that had between 8 and 11 years of schooling.^9,10^ The above-mentioned international studies examining the test-retest reliability of the OTI do not report on variables such as duration of substance use or medical comorbidities such as HIV; therefore, it is difficult to ascertain whether the differences in results noted in our sample were impacted by these factors. Within our sample of nyaope users, the test-retest reliability differed with regard to specific substances.

Interestingly, with the exception of tobacco, most of the substances that had excellent ICC reliability were used by fewer participants and used less frequently compared to the poorer reliability for more frequently used substances. For example, only eight participants used crystal methamphetamine and the average use of it was more than once a week compared to nyaope, which was used daily by all of the participants. It may be that participants had a more accurate recall of the number of past month episodes for substances that were less frequently used. Recall of tobacco was, however, an exception, in that it was used frequently but demonstrated a high test-retest reliability. This may be because of tobacco being legal and more acceptable to use, which may have encouraged users to report its use more truthfully and freely without fear of stigma. In this sample, the test-retest correlation of other opiates was 1.00. For inhalants, the ICC was 0.00 and the Brennan–Prediger coefficient 0.96, but this must be interpreted with caution as only two participants reported using other opiates and one reported using inhalants.

Importantly, there was poor reliability of the Q-scores of cannabis, nyaope, alcohol and methaqualone. There are several potential reasons for this: one being that the questions asked in order to calculate the Q-scores (frequency of past month substance use) require the user to state the exact number of times the substance was used per day in the last 3 days the substance was used. This may influence the accuracy and consistency of responses as the answer depends on the inherent reliability of the substance user to self-report substance use and the ability of the user to accurately recall the exact number of episodes of substance use. Another factor to consider is the inherent reliability of self-report research tools in the field of addiction medicine.

Demetriou describes some of the reasons why self-reporting may be unreliable. Demetriou explains some of the factors impacting self-reported substance use.^24^ The first being that participants tend to deliberately misinform or underreport certain facts.^24,25,26^ This may be because of concerns about social desirability and stigma associated with substance use. Other reasons include familiarity with the interviewer, which also contributes to whether the participant will answer truthfully or falsely. Confidentiality and the fear that the responses to questions of a sensitive nature may be later divulged to third parties, such as treating doctors or rehabilitation centres, have also been cited as a barrier to transparent self-reporting. Cultural barriers such as the phrasing of questions also impact responses on self-report questionnaires.^27^ Kilian et al. found that a language barrier and informal interpretation carried out by untrained professionals can impact the outcomes of mental health evaluations, through over or underdiagnosing certain conditions.^27^ When comparing self-reported substance use to saliva, urine or blood laboratory analysis, literature often shows that self-report can be reliable, however, should be used in conjunction with toxicology.^26,28,29^

Finally, the requirement of recalling specific details may be challenging for substance users because of confounding variables such as substance-induced neurocognitive deficits.^30^ In a number of studies, chronic substance use has been negatively associated with impaired cognitive functioning, viz. memory impairment. These studies report that memory may be irreversibly impaired in users who have been using heroin chronically.^30,31^ In our study, cognitive deficits as a result of heroin use, polysubstance use or HIV may have impacted the participants’ ability to accurately remember and report the number of episodes of substances used.^30,31^A recently published study compared the magnetic resonance imaging (MRI) findings of 30 nyaope users to healthy controls. The study reported significant grey matter fronto-temporal cortical atrophy in regions involved in working memory in nyaope users.^32^ These MRI findings may support an association between poor test-retest reliability and impaired cognition in our study population. Murphy et al.^33^ notes that memory can still be irregular and inconsistent in self-reporting, even without substance use. So variability in reporting details about substance use quantities, because of memory impairments, should always be expected even without a history of substance use. It is suggested that a baseline cognitive screening tool be used to assess for possible memory impairments, for measures to be put in place in those participants who show signs of impairment in order to mitigate their effects on recall.^30,33,34^ Taking all these factors into consideration, it is interesting and surprising to observe that in this study there was excellent reliability for almost all substances when assessing the variable of any past month use versus past month abstinence.

Using the Brennan–Prediger coefficient to assess substance use, all substances showed almost perfect agreement, with the exception of alcohol that had substantial agreement. The difference in results between the test-retest reliability of frequency of substance use and the test-retest reliability of only reporting use versus no use may suggest that within this sample, users were reliable in stating that they used a substance, however, were not as reliable in recalling how often the substance was used. The excellent test-retest reliability using use versus abstinence is thought to be because of the dichotomous nature of answers provided. No recall of specific details was required, only a yes or no answer about substance use in the past month. The reliability of other domains of the OTI is a further positive finding of this study.

### Limitations

There are limitations to this study that may have impacted the results. The majority of the participants were male, with very few females; thus these findings may not be generalisable to females. In addition, all the individuals recruited were being treated as inpatients. It would have been of great of value to recruit individuals receiving outpatient substance use treatment and to compare the findings with those who were inpatients. The most significant limitation is that the study only investigated the test-retest reliability of the OTI and did not do a comprehensive assessment of all aspects of reliability or validity.

## Conclusion

When measuring the test-retest reliability of the OTI in a sample of nyaope users, it was observed that some domains performed better than others. The past month drug-use section of the OTI, which aims to assess the frequency of substances used in the past month, performed poorly for nyaope, which was the primary drug of choice. Although this study found that there was an excellent correlation between interviews for unquantified self-reported substance use, the drug use section of the OTI was not created to determine this variable. Other methods of assessing substance use, for example, blood, urine and hair follicle testing, should accompany the drug use section of the OTI.

A significant finding was the excellent test-retest reliability when participants self-reported on injecting and sexual behaviour and moderate reliability of the criminality, general health and social functioning domains. High-risk injecting and sexual behaviour are of significant concern among heroin users, especially in the South African context of HIV. It is also vitally important to evaluate treatment outcomes of heroin users holistically, and thus these preliminary findings suggest that these sections of the OTI may have research utility in South Africa. However, further studies into the full reliability and validity of the OTI in South Africa are needed. There were several limitations to the scope of the study; therefore, unequivocal conclusions cannot, at this stage, be drawn about the complete utility of the tool in a South African setting.
